# Integrin‐Binding Matricellular Protein Fibulin‐5 Maintains Epidermal Stem Cell Heterogeneity During Skin Aging

**DOI:** 10.1111/acel.70483

**Published:** 2026-04-10

**Authors:** Wenxin Fan, Mizuho Ishikawa, Erna Raja, Ahmed M. Hegazy, Hinata Date, Yen Xuan Ngo, Yoshifumi Sato, Kazuya Yamagata, Hiromi Yanagisawa, Aiko Sada

**Affiliations:** ^1^ International Research Center for Medical Sciences (IRCMS) Kumamoto University Kumamoto Japan; ^2^ Division of Skin Regeneration and Aging, Medical Institute of Bioregulation Kyushu University Fukuoka Japan; ^3^ Graduate School of Medical Sciences Kumamoto University Kumamoto Japan; ^4^ Life Science Center for Survival Dynamics, Tsukuba Advanced Research Alliance (TARA) University of Tsukuba Tsukuba Japan; ^5^ Zoology Department, Faculty of Science Minia University El‐Minia Egypt; ^6^ Department of Medical Biochemistry, Faculty of Life Sciences Kumamoto University Kumamoto Japan; ^7^ Center for Metabolic Regulation of Healthy Aging (CMHA), Faculty of Life Sciences Kumamoto University Kumamoto Japan; ^8^ Faculty of Medicine University of Tsukuba Tsukuba Japan

**Keywords:** epidermal stem cells, extracellular matrix, fibulin‐5, integrins, skin aging, YAP

## Abstract

The extracellular matrix (ECM) is crucial in building the extracellular environment and translating extracellular information into biochemical signals that sustain tissue functions. Fibulin‐5 (*Fbln5*) is a multifunctional ECM protein essential for forming elastic fibers and regulating cellular functions by binding to integrins. Although fibulin‐5 expression decreases with age in human skin, its functional implications, particularly in epidermal stem cell regulation, remain largely unexplored. Here, we show that the loss of *Fbln5* in mice leads to early impairments of epidermal stem cell properties that resemble changes observed during chronological skin aging. *Fbln5* deficiency is associated with reduced expression of integrins and other cell junction proteins and decreased YAP activation in epidermal stem cells. Pharmacological inhibition of YAP reduces the fast‐cycling stem cell region in mice and downregulates the fast‐cycling epidermal stem cell marker SLC1A3 in human primary keratinocytes. At the cellular level, YAP activity and SLC1A3 expression are modulated by cell density, with low‐density cultures exhibiting high nuclear YAP and elevated SLC1A3 expression, whereas high‐density conditions suppress both. Under high‐density conditions, fibulin‐5 coating partially restores nuclear YAP localization and increases SLC1A3 expression. Together, these findings suggest that, beyond its structural role in elastic fiber formation, fibulin‐5 contributes to the maintenance of epidermal stem cell balance during skin aging by linking extracellular alterations to YAP‐dependent intracellular signaling.

## Graphical abstract

Fibulin‐5 maintains epidermal stem cell heterogeneity during skin aging by linking the extracellular environment to YAP‐dependent intracellular signaling. Its loss reduces YAP activity and depletes the fast‐cycling epidermal stem cell population, recapitulating age‐associated changes in skin.

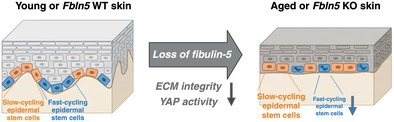

## 1 Introduction

The interfollicular epidermis, the outermost layer of the skin, functions as a protective barrier that is maintained by lifelong self‐renewal and differentiation of epidermal stem cells. Recent single‐cell and lineage analyses suggest that these stem cell populations are heterogeneous in their molecular properties and cellular lineages (Ghuwalewala et al. 2022; Joost et al. 2016; Koren et al. 2022; Mascré et al. 2012; Rognoni and Watt 2018; Wang et al. 2020). Notably, mouse tail skin exhibits interscale and scale structures that are maintained by slow‐ and fast‐cycling epidermal stem cell populations characterized by Dlx1 and Slc1a3, respectively (Gomez et al. 2013; Sada et al. 2016). During skin aging, there is a gradual loss of Slc1a3^+^ fast‐cycling epidermal stem cells, resulting in an imbalance of the epidermal stem cell population (Raja et al. 2022). Similar epidermal stem cell heterogeneity has been observed in human skin, with their localization corresponding to the undulating structures known as rete ridges and inter‐ridges (Ghuwalewala et al. 2022; Lawlor and Kaur 2015). However, the factors regulating epidermal stem cell heterogeneity in aging skin remain poorly understood.

The extracellular matrix (ECM) provides both a biochemical and mechanical environment for tissue stem cells. In the skin, the ECM includes the dermal compartment, which is primarily composed of collagen types I and III and elastic fibers, and the basement membrane zone at the epidermal–dermal junction (Chermnykh et al. 2018; Raja et al. 2023). Integrins, hemidesmosomes, and other cell junctional molecules anchor epidermal stem cells to the basement membrane, maintaining their undifferentiated state (Liu et al. 2019; Wang et al. 2022; Watt 2002). Integrins transduce extracellular cues into intracellular signaling pathways that influence epidermal stem cell function (AlDahlawi et al. 2006; Morgner et al. 2015; Watt 2002). Deleting integrin β1 in mice leads to a loss of the parakeratotic scale area (López‐Rovira et al. 2005), and aged human skin shows reduced integrin β1 expression in epidermal stem cells (Giangreco et al. 2010). Additionally, levels of fibulin‐7 (*Fbln7*), a matricellular protein at the basement membrane, decrease in aged skin, and *Fbln7* knockout (KO) results in the gradual depletion of fast‐cycling epidermal stem cell clones (Raja et al. 2022). Changes in ECM composition and structure alter the mechanical environment for hair follicle and epidermal stem cells, thereby modulating tissue and cellular functions (Aragona et al. 2020; Ichijo et al. 2022; Koester et al. 2021; Li et al. 2022; Xie et al. 2022). However, the factors coordinating the extracellular environment and intracellular signals to regulate epidermal stem cell heterogeneity remain unclear.

Fibulin‐5 (*Fbln5*) is a structural component of elastic fiber networks (Yanagisawa et al. 2009). In the skin, fibulin‐5 is localized in the papillary dermis, oriented perpendicular to the epidermis, and integrates with other elastic fiber components in the reticular dermis (Kadoya et al. 2005). During aging, fibulin‐5 expression decreases, particularly in the papillary dermis region (Kadoya et al. 2005; Langton et al. 2019). *Fbln5* KO mice show defects in elastic fiber formation, resulting in skin, arterial, and lung abnormalities (Yanagisawa et al. 2002). In individuals with cutis laxa, *FBLN5* is associated with the development of loose and sagging skin, resembling the appearance of premature aging (Gharesouran et al. 2021). Fibulin‐5 regulates tissue stiffness and the inflammatory response in skin fibrosis (Nakasaki et al. 2015). In addition to its role in elastic fiber formation, fibulin‐5 can also interact with integrins, including α5β1, αvβ3, and αvβ5, via its arginine–glycine–aspartic acid (RGD) motif (Budatha et al. 2011; Lomas et al. 2007; Nakamura et al. 2002; Yanagisawa et al. 2009). These integrin interactions have been implicated in cell–ECM adhesion and mechanosensitive cellular responses (Roca‐Cusachs et al. 2009). This study investigated how the age‐dependent decrease in fibulin‐5 modulates the extracellular environment and influences the cellular and molecular properties of epidermal stem cells during skin aging.

## 2 Materials and Methods

### 2.1 Mice and Ethics

Experimental mice were housed in specific pathogen‐free facilities in the Center for Animal Resources and Development (CARD) at Kumamoto University, the Laboratory of Embryonic and Genetic Engineering at Kyushu University, and the Laboratory Animal Resource Center at the University of Tsukuba. The mice were maintained under a 12/12‐h light/dark cycle at a controlled temperature of 22°C ± 1°C–2°C, with free access to water and standard chow. C57BL/6J mice were obtained from Charles River Laboratories Japan Inc. (Kanagawa, Japan) and Japan SLC Inc. (Shizuoka, Japan). All animal procedures were conducted according to the institutional animal care guidelines of each university and were approved by the respective animal research and ethics committees. Mice of both sexes were used in all animal experiments and were randomly allocated to experimental groups.

The generation of *Fbln5* KO mice (129SvEv; C57BL/6) was reported previously (Okuyama et al. 2017). BrdU (5‐bromo‐2′‐deoxyuridine; Sigma‐Aldrich) was administered in drinking water (0.8 mg/mL) 2 days before sacrifice to label cells in the S‐phase of the cell cycle.

For the YAP inhibitor verteporfin treatment, 2–3‐month‐old C57BL/6J mice received subcutaneous injections of verteporfin (Selleck Chemicals; 1 mg/mL in 5% DMSO/PBS) or an equivalent volume of vehicle control (5% DMSO/PBS). A total volume of 60 μL was injected at two sites into the tail skin per mouse. Injections were administered twice at 3‐day intervals. Mice were euthanized 3 days after the final injection, and skin tissues were collected for subsequent analyses.

### 2.2 Human Skin Samples and Ethics

Frozen full‐thickness normal human abdominal skin samples embedded in optimal cutting temperature (OCT) compound were purchased from CTI‐Biotech (Lyon, France) under ethical approval and in compliance with applicable ethical regulations. Samples were grouped by age as 20–40 years (SK0163, SK548, SK550) and > 50 years (PK003, SK0033, SK220). Detailed donor information, including age, sex, ethnicity, and skin site, is provided in Table S1. Informed consent was obtained from the donors by the supplier, and the samples were anonymized prior to purchase.

### 2.3 Whole‐Mount Immunostaining

Whole‐mount staining of mouse tail skin was performed as previously described (Sada et al. 2016). The following primary antibodies were used: rat anti‐BrdU (1:300, Abcam, ab6326), mouse anti‐K10 (1:100; Abcam, ab9026), guinea pig anti‐K36 (1:100; Proteintech, 14309‐1‐AP), and rat anti‐Ki‐67 (1:100; eBioscience [Invitrogen], 14‐5698‐82). A mouse‐on‐mouse kit (Vector Laboratories) was used to detect the primary mouse antibody. Before incubation with the anti‐BrdU antibody, the samples were treated with 2 N HCl at 37°C for 30 min. Following primary antibody incubation and washing, samples were incubated with species‐specific Alexa Fluor‐conjugated secondary antibodies (488, 555, or 647; 1:500; Thermo Fisher Scientific) for 1 h at room temperature. All samples were stained with Hoechst (Sigma‐Aldrich) before mounting. Whole‐mount epidermis samples were imaged using Z‐stack acquisition under a confocal microscope (Nikon A1 HD25 or Zeiss LSM900). Z‐series were acquired with identical settings within each experiment using NIS Elements Imaging (Nikon) or Zen 3 (Blue Edition) (Carl Zeiss) software, and maximum intensity projections were generated for analysis. All images are presented as projected Z‐stacks viewed from the basal layer side.

### 2.4 Hematoxylin and Eosin Staining and Section Immunostaining

Mouse tail skin was directly embedded in OCT compound (Sakura Fine Tec). The 10 μm sections were fixed in 4% paraformaldehyde at room temperature (RT) for 10 min. Next, the sections were stained with hematoxylin (Wako) for 20 min and eosin Y (Wako) for 15 s before dehydration and mounting in Entellan solution (Merck Millipore). Images were captured using an EVOS M5000 Imaging System (Thermo Fisher Scientific).

Skin sections were immunostained as previously described (Sada et al. 2016). The rabbit anti‐fibulin‐5 antibody (BSYN1923) was reported previously (Yanagisawa et al. 2002). Specificity of the anti‐fibulin‐5 antibody was confirmed using *Fbln5* KO skin as a negative control. The following primary antibodies were used: rabbit anti‐collagen XVII (1:300; Abcam, ab184996), rat anti‐integrin α6 (1:150; BD Biosciences, 555734), rabbit anti‐integrin β1 (1:200; Cell Signaling Technology, 34971S), rabbit anti‐integrin β3 (1:100; Thermo Fisher Scientific, MA5‐32077), chicken anti‐K5 (1:500; BioLegend, 905904), rat anti‐nectin‐3 (1:100; Abcam, ab16913), guinea pig anti‐K36 (1:100; Proteintech, 14309‐1‐AP), rabbit anti‐YAP (1:100; Cell Signaling Technology, 4912S), rabbit anti‐ASS1 (1:1000; Cell Signaling Technology, 05‐665), and guinea pig anti‐SLC1A3 (1:100; Frontier Institute, GLAST‐GP‐Af1000). Following primary antibody incubation and washing, sections were incubated with species‐specific Alexa Fluor‐conjugated secondary antibodies (488, 555, or 647; 1:300; Thermo Fisher Scientific) for 1 h at RT. The sections were counterstained with Hoechst (Sigma‐Aldrich) for 10 min. The sections were imaged using a confocal microscope (Nikon A1 HD25 or Zeiss LSM900). Laser power and detector gain were kept constant across age groups within each experiment. The image brightness and contrast were adjusted linearly and uniformly using Adobe Photoshop (Adobe Inc.).

### 2.5 Fluorescence‐Activated Cell Sorting (FACS)

Subcutaneous and fat tissues were removed from the tail skin and incubated in 0.25% trypsin/EDTA overnight at 4°C and then at 37°C for 30 min the next day. Single‐cell suspensions were prepared by gentle scraping of the epidermis. Cells were stained with the following antibodies for 30 min on ice: biotin‐conjugated CD34 (1:50, eBioscience, 13‐0341‐85), APC‐conjugated streptavidin (1:100; BD Biosciences, 554067), BV510‐conjugated integrin α6 (1:200; BD Biosciences, 563271), and BV421‐conjugated Sca‐1 (1:100; BD Biosciences, 562,729). Dead cells were excluded by 7‐AAD staining (BD Biosciences). Epidermal basal cells were gated on the expression of integrin α6, CD34, and Sca‐1, as shown in Figure 2A, and the same gating strategy was applied across all age groups. Cells were isolated using a FACSAria flow cytometer (BD Biosciences). The data were analyzed using FlowJo software (BD Biosciences).

### 2.6 RNA‐Sequencing (RNA‐Seq) Analysis

The FACS‐isolated cells from *Fbln5* wild‐type (WT) and KO mice (*n* = 4 per group) were directly sorted into TRIzol LS Reagent (Ambion) and processed by Tsukuba i‐Laboratory LLP at the University of Tsukuba. RNA concentration and purity were assessed using a NanoDrop spectrophotometer and an Agilent 2100 Bioanalyzer (RNA 6000 Pico Kit). RNA‐seq libraries were prepared using the SMARTer Stranded Total RNA‐Seq Kit v2—Pico Input Mammalian (Takara) and sequenced on an Illumina NextSeq 500 platform. Sequencing reads were mapped to the mouse reference genome with gene annotation using CLC Genomics Workbench (Qiagen). Gene‐level expression values were obtained as raw read counts. Raw read counts were normalized across samples using quantile normalization implemented in CLC Genomics Workbench. Differential expression analysis between WT and KO samples was performed using the statistical analysis module in CLC Genomics Workbench. Fold‐change analysis was performed using log_2_‐transformed normalized expression values, and genes exhibiting ≥ 2‐fold change were selected for downstream analyses.

Principal component analysis (PCA) was performed using CLC Genomics Workbench. Gene Ontology (GO) enrichment analysis was conducted using Metascape (https://metascape.org) with genes exhibiting ≥ 2‐fold change. Volcano plots were generated by plotting the log_2_‐fold change against the –log_10_ (*p*‐value) using SRplot (Tang et al. 2023). Genes were considered differentially expressed if they showed an absolute fold change ≥ 2 and a *p* < 0.05. Hierarchical clustering and heatmap visualization were performed using ClustVis (https://biit.cs.ut.ee/clustvis/). Quantile‐normalized expression values were log_2_‐transformed for clustering and visualization. To avoid undefined values during log transformation, a pseudocount of 0.01 was added prior to transformation. For visualization of individual gene expression levels, normalized RPKM (reads per kilobase of transcript per million mapped reads) values calculated in CLC Genomics Workbench were used.

### 2.7 Cell Culture

Neonatal primary human keratinocytes (KER112002, KER112005, and KER112006; Biopredic International) were cryopreserved in CnT‐Cryo‐50 (CELLnTEC), and cells with fewer than 10 passages were used in the experiments. After thawing, keratinocytes were cultured in 100 mm dishes (7 mL medium/dish) coated with collagen type IV (50 μg/mL in PBS; Sigma‐Aldrich) in CnT‐Prime medium (CELLnTEC) at 37°C in a humidified atmosphere with 5% CO_2_. Once the cells reached 80%–90% confluency, they were passaged using Accumax (Innovative Cell Technologies) and Accutase (Innovative Cell Technologies).

For the cell density experiments, primary human keratinocytes were seeded at 150,000, 50,000, and 25,000 cells/well in 12‐well plates coated with collagen type IV (50 μg/mL in PBS). Cells were cultured in CnT‐Prime medium for 48 h post‐seeding under standard culture conditions. Samples were fixed with 4% paraformaldehyde at RT for 10 min before staining. Immunofluorescence staining was performed as previously described (Xuan Ngo et al. 2021) using the following primary antibodies: rabbit anti‐YAP (1:100; Cell Signaling Technology, 4912S), mouse anti‐Ki‐67 (1:200; BD Bioscience, 14‐5698‐82), rabbit anti‐ASS1 (1:1000; Cell Signaling Technology, 05‐665), and guinea pig anti‐SLC1A3 (1:100; Frontier Institute, GLAST‐GP‐Af1000).

For YAP inhibition assays, primary keratinocytes were seeded at 50,000 cells per well in 12‐well plates coated with collagen type IV (50 μg/mL in PBS) and cultured for 24 h before treatment. Cells were treated with verteporfin (5 μg/mL in 0.07% DMSO) or vehicle control (0.07% DMSO) for 8 h prior to fixation or RNA extraction. The verteporfin dose was determined based on previous studies (Huang et al. 2022).

For the fibulin‐5 coating assay, the 12‐well plates were coated overnight at 4°C with collagen type IV (50 μg/mL in PBS) either alone or with 90 ng/mL recombinant human fibulin‐5 (R&D Systems) in PBS. Primary keratinocytes (300,000 cells per well) were seeded on these plates and cultured to ~80% confluence. The medium was then replaced, and cells were harvested 8 h later for fixation or RNA extraction.

### 2.8 RNA Isolation and RT‐qPCR

For mouse epidermal basal cells isolated by FACS, cells were directly sorted into TRIzol LS Reagent. Following chloroform extraction, the aqueous phase was collected, and total RNA was purified using the RNeasy Micro Kit (Qiagen) according to the manufacturer's instructions. For RNA extraction from cultured primary human keratinocytes, cells were lysed directly in culture wells with Buffer RLT supplemented with β‐mercaptoethanol. Cells were then collected by scraping, and total RNA was isolated using the RNeasy Mini Kit according to the manufacturer's instructions.

cDNA was synthesized using an iScript cDNA Synthesis Kit (Bio‐Rad). RT‐qPCR was conducted on a LightCycler 96 System (Roche) using the FastStart Essential DNA Green Master (Roche). Relative gene expression levels were calculated using the ΔΔCt method and normalized to housekeeping genes. A complete list of primers used for mouse and human samples is provided in Table S2.

### 2.9 Quantification and Statistical Analysis

All quantifications were performed independently using at least three independent biological replicates. The data are presented as the mean ± standard deviation (SD). Statistical analyses were performed using GraphPad Prism 9 (GraphPad Software), and the statistical tests used are stated in the corresponding figure legends. A *p*‐value < 0.05 was considered statistically significant. Statistical significance was defined as follows: *p < 0.05; **p < 0.01; ***p < 0.001; ****p < 0.0001; ns, not significant. Animals were randomly allocated to experimental groups where applicable. Investigators were not blinded during data acquisition or analysis.

**FIGURE 1:**
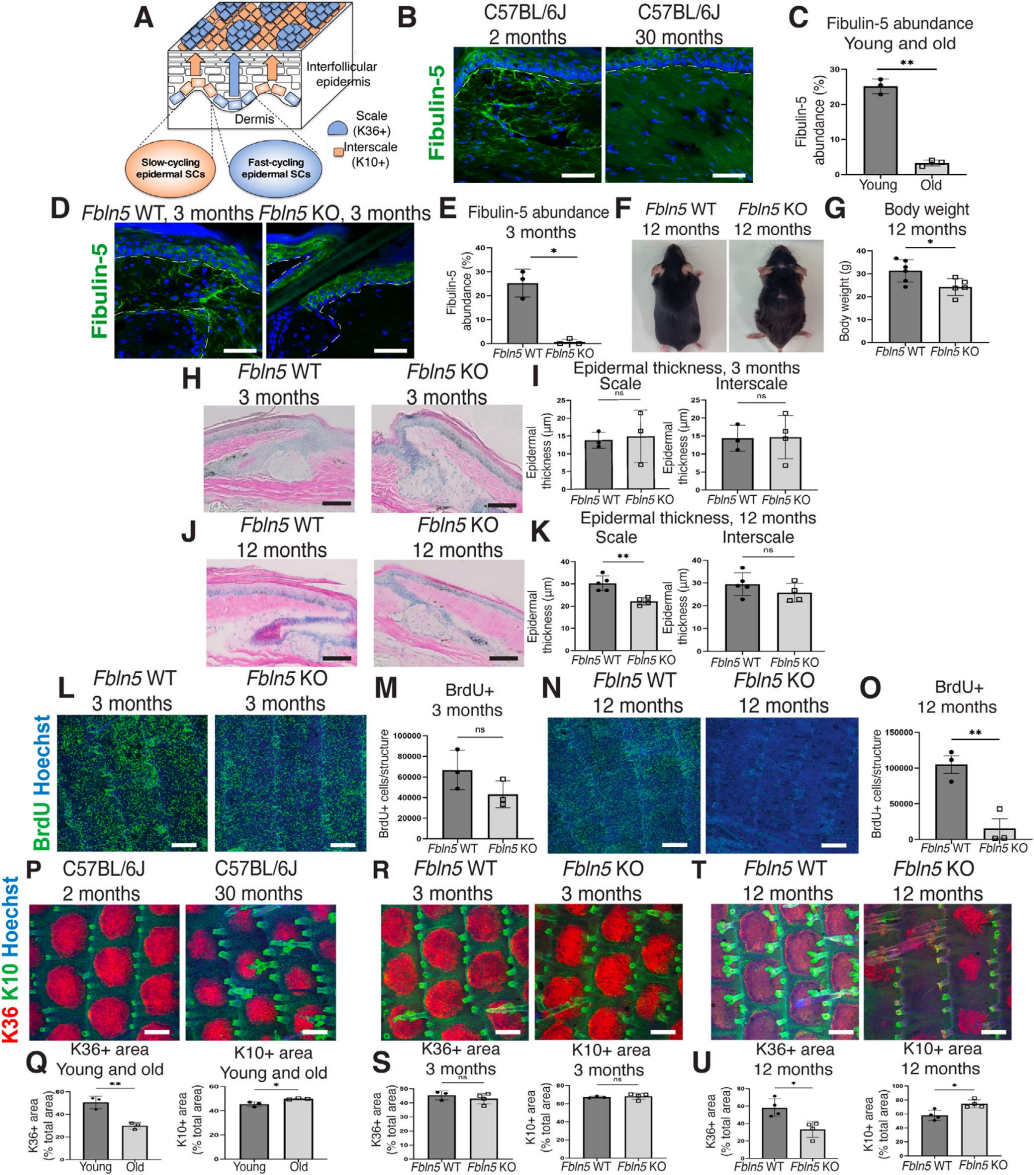
Impact of fibulin‐5 deficiency on the skin aging process. (A) Schematic representation of the interfollicular epidermis of mouse tail skin. Slow‐cycling epidermal stem cells (SCs) produce the K10^+^ interscale lineage (orange), and fast‐cycling epidermal SCs produce the K36^+^ scale lineage (blue). (B, C) Immunostaining of fibulin‐5 (green) in sections of mouse tail skin from 2‐month‐old versus 30‐month‐old C57BL/6J mice and quantification (C). The white dashed line represents the epidermal–dermal boundary. Scale bars: 50 μm. (D, E) Immunostaining of fibulin‐5 (green) in sections of mouse tail skin from 3‐month‐old *Fbln5* WT versus KO mice and quantification (E). The white dashed line represents the epidermal–dermal boundary and hair follicles. Scale bars: 50 μm. (F) Images of 12‐month‐old *Fbln5* WT and KO mice. (G) The body weights of 12‐month‐old *Fbln5* WT and KO mice. (H–K) Hematoxylin and eosin staining of sagittal sections of the skin of 3‐ and 12‐month‐old *Fbln5* WT versus KO mice and quantification (I, K). Scale bars: 150 μm. Epidermal thickness was measured in interscale and scale regions. (L–O) Whole‐mount staining of BrdU (green, a proliferation marker) and Hoechst (blue) in 3‐ and 12‐month‐old *Fbln5* WT versus KO mice and quantification (M, O). Scale bars: 200 μm. (P–U) Whole‐mount staining of K10 (green, interscale lineage), K36 (red, scale lineage), and Hoechst (blue) in 2‐month‐old versus 30‐month‐old C57BL/6J mice and 3‐ and 12‐month‐old *Fbln5* WT versus KO mice and quantification (Q, S, U). Scale bars: 200 μm. All data are presented as the mean ± SD. Each dot represents one mouse. Statistical significance is assessed using a two‐tailed unpaired *t*‐test (C, E, G, I, K, M, O, Q, S, U). *, *p* < 0.05; **, *p* < 0.01; ns, not significant.

Quantification of fluorescence intensity and area measurements was performed using ImageJ (NIH). The abundance (%) of fibulin‐5 antibody staining was quantified (Figure 1C,E) using a previously described method (Langton et al. 2019). Briefly, the positively stained area was quantified relative to the total tissue region of interest (ROI) area. The percentage of positive area was calculated as: abundance (%) = (positively stained area/total ROI area) × 100. In tail sections, an epidermal unit was defined as the interfollicular epidermis region comprising one scale and one interscale structure between two hair follicles (Changarathil et al. 2019). The epidermal thickness was measured for at least six epidermal units per mouse (Figure 1I,K; Figure S1B). BrdU^+^ and Ki‐67^+^ cells were manually counted in 4–8 projected Z‐stack images per mouse, each containing 15–20 interscale or scale structures (Figure 1M,O; Figure S1D,F). K10^+^ and K36^+^ areas were measured in 6–8 projected Z‐stack images per mouse, each containing 30 interscale or scale structures (Figure 1Q,S,U; Figure 4N; Figure S1H,J). The integrated intensity of collagen XVII, integrin β1, integrin α6, and integrin β3 was measured in epidermal basal cells, excluding nuclear signals (Figure 3D,F,H,J,L,N,P,R; Figure S2D). At least 50 cells were analyzed for each sample. The length of the nectin‐3^+^ area (Figure 3T,V; Figure S2G) and the overlap ratio between nectin‐3 and K36 were measured (Figure S2H,I). The YAP, Ki‐67, SLC1A3, and ASS1 signals were calculated using the maximum intensities of the nucleus regions and regions adjacent to the nucleus (Figure 4C,E,I,L; Figure 5B,D,F,H,J,O). More than 100 cells were measured per biological replicate.

For quantification of ASS1 and SLC1A3 fluorescence intensity in human skin sections (Figure 4G), images were analyzed using ImageJ. In young samples (20–40 years), the distance from the lowest point of the rete ridge structure to the target cells along the basement membrane was measured. Based on this distance, basal cells were categorized into two spatial compartments: 0–40 μm (rete ridge region) and > 40 μm (inter‐ridge region). In old samples (> 50 years), where rete ridge structures were markedly flattened, spatial subdivision was not applied, and the entire basal layer was used for quantification. For each basal cell, the integrated fluorescence intensity of ASS1 or SLC1A3 was measured, and the background signal was estimated from the nuclear region of the same cell and subtracted from the total signal to obtain the corrected fluorescence intensity. For each donor, multiple non‐overlapping fields were analyzed (at least five images per sample), and at least 50 basal cells were quantified per donor. The mean corrected fluorescence intensity was calculated for each region (young samples) or for the entire basal layer (old samples). Three independent donor samples were analyzed per age group.

## 3 Results

### 3.1 *Fbln5* KO Mice Show Early Impairments Resembling Epidermal Stem Cell Aging

To examine age‐related changes in fibulin‐5 expression in mice, we performed immunostaining on 2‐month‐old (young) and 30‐month‐old (old) C57BL/6J mice. In young skin, fibulin‐5 was observed around the hair follicles in the dermis and exhibited candelabra‐like structures below the interfollicular epidermis (Figure 1A–E). The absence of dermal signals in *Fbln5* KO mice confirmed the specificity of the fibulin‐5 antibody (Figure 1D,E). Signals were also detected in the epidermis; however, similar signals were observed in *Fbln5* KO mice (Figure 1D), indicating that the epidermal staining likely represents non‐specific background. We observed a decrease in fibulin‐5 levels in aged skin, particularly in the papillary dermis region below the interfollicular epidermis (Figure 1B,C), reflecting the age‐dependent decline reported in human skin (Kadoya et al. 2005; Langton et al. 2019).

To assess the impact of fibulin‐5 deletion, we analyzed the skin phenotypes of *Fbln5* KO mice. As reported previously, young *Fbln5* KO mice exhibited skin laxity (Yanagisawa et al. 2002). By 12 months of age, both male and female *Fbln5* KO mice exhibited additional gross phenotypes, including a brownish coat, thinner hair, and lower body weight than WT mice (Figure 1F,G). Histologically, epidermal thickness did not differ significantly between mice aged 3 or 6 months (Figure 1H,I; Figure S1A,B); however, the epidermis became significantly thinner in the *Fbln5* KO mice compared with the *Fbln5* WT mice by 12 months (Figure 1J,K). The proliferation of epidermal stem cells also did not differ between 3‐ and 6‐month‐old *Fbln5* KO mice but was significantly reduced in 12‐month‐old mice compared with age‐matched control mice (Figure 1L–O; Figure S1C–F). These phenotypes are consistent with the previously reported age‐related skin atrophy (Changarathil et al. 2019).

To further examine the changes in epidermal stem cells, we examined the dynamics of epidermal stem cells throughout development and aging. The interscale and scale structures in tail skin are formed at the neonatal stage by 2 weeks of age, which coincides with the establishment of epidermal stem cell heterogeneity (Dekoninck et al. 2020; Gomez et al. 2013; Sada et al. 2016). In young adult mice, these structures maintain a constant size, replenished by slow‐ and fast‐cycling epidermal stem cells (Sada et al. 2016). However, the size of the scale regions was significantly reduced in chronologically aged mice (Figure 1P,Q), consistent with the loss of the fast‐cycling epidermal stem cell population (Raja et al. 2022).

In *Fbln5* KO mice, the interscale and scale sizes were unaffected at the neonatal stage (Figure S1G,H) and in young adults (3 and 6 months; Figure 1R,S; Figure S1I,J). However, by 12 months, while WT mice retained normal interscale/scale size, *Fbln5* KO mice exhibited a significant decrease in scale size (Figure 1T,U). These results suggest that the loss of *Fbln5* does not significantly affect epidermal stem cell development and homeostasis at a younger age. However, its effects become more pronounced with aging, possibly due to chronic inflammation or interactions with other ECM components.

### 3.2 *Fbln5* KO Mice Exhibit Molecular Changes Indicative of Epidermal Stem Cell Aging

**FIGURE 2:**
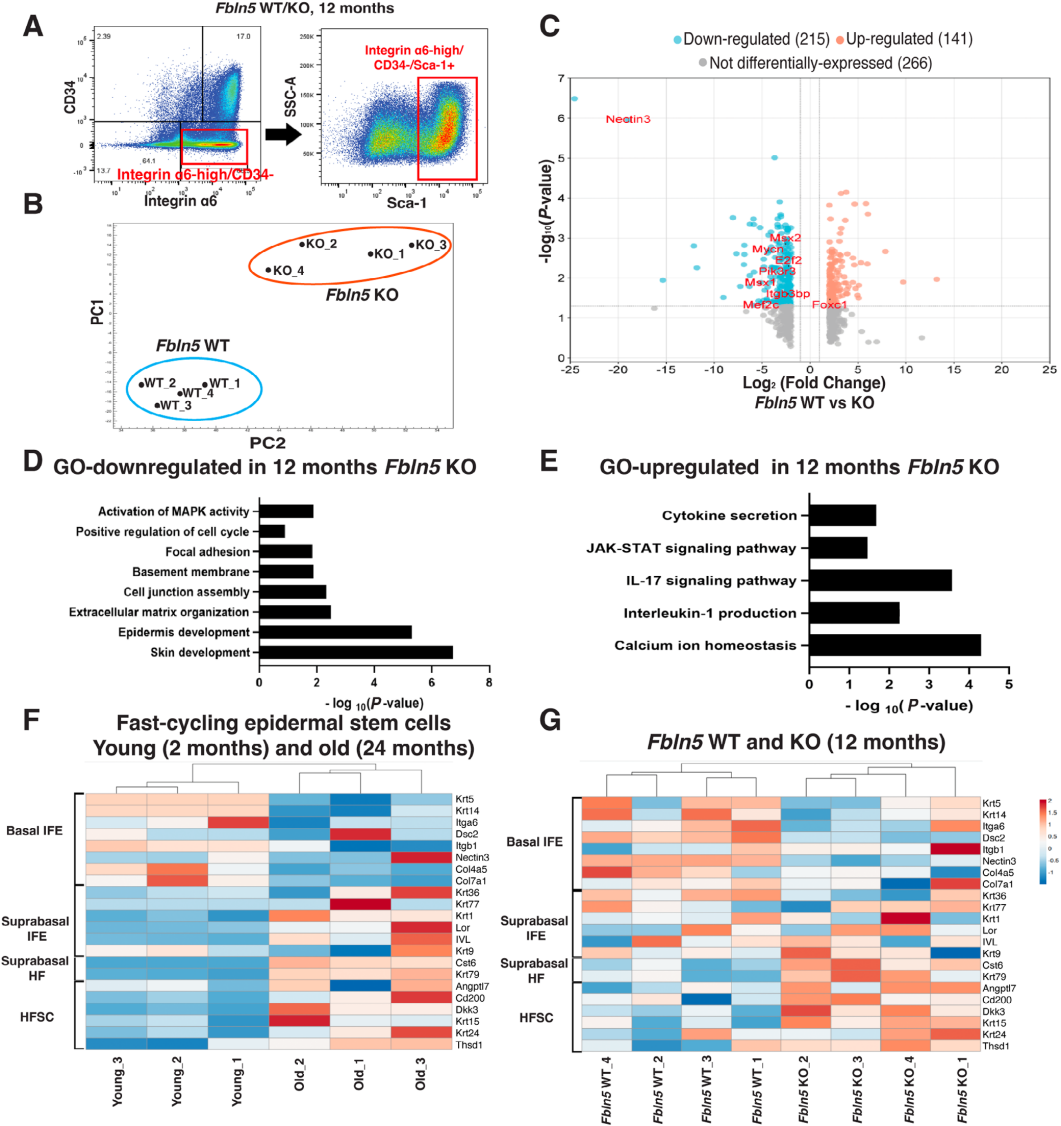
Transcriptome analysis of epidermal stem cells in *Fbln5*‐deficient mice. (A) Representative FACS plots. Integrin α6^high^/CD34^−^/Sca1^+^ cells were isolated as epidermal stem cells. (B) Principal component analysis of genes with a ≥ 2‐fold change in 12‐month‐old *Fbln5* WT and KO mice. Each dot represents one mouse (*n* = 4/group). (C) Volcano plots showing differentially expressed genes in 12‐month‐old *Fbln5* WT and KO mice. (D, E) Gene ontology analysis of genes with ≥ 2‐fold downregulation (D) or upregulation (E) in *Fbln5* KO versus WT mice. (F, G) The heatmaps show basal and suprabasal signature genes of the interfollicular epidermis and hair follicle lineages, based on RNA‐seq data from young (2‐month‐old) and old (24‐month‐old) fast‐cycling epidermal stem cells and 12‐month‐old *Fbln5* WT and KO epidermal stem cells.

To explore the molecular changes in epidermal stem cells induced by the loss of *Fbln5*, we performed RNA‐seq analysis of FACS‐isolated epidermal stem cells from *Fbln5* WT and KO mice at 12 months of age (Figure 2A). Principal component analysis revealed global transcriptome changes between *Fbln5* WT and KO mice (Figure 2B). We identified differentially expressed genes (DEGs) that were significantly upregulated or downregulated in *Fbln5* KO epidermal stem cells (Figure 2C; Table S3). To validate the RNA‐seq data, we selected representative downregulated genes associated with cell cycle regulation (*E2f2*, *Cdk1*) and cell adhesion (*Nectin3*) and performed RT‐qPCR analysis. The expression levels of these genes were significantly reduced in *Fbln5* KO compared with WT controls (Figure S2A), confirming the reliability of the transcriptomic analysis.

Gene Ontology analysis revealed that the downregulated DEGs were enriched for genes related to the cell cycle, the MAPK signaling pathway, focal adhesion, ECM/basement membrane, and skin development (Figure 2D). The upregulated DEGs were enriched for genes related to pathways associated with cytokine production, the JAK–STAT signaling pathway, and calcium ion homeostasis (Figure 2E). These gene changes have been reported as signatures of aged epidermal stem cells (Ichijo et al. 2022; Raja et al. 2022).

To assess similarities with chronologically aged skin, we compared the 12‐month‐old *Fbln5* WT/KO datasets with previously characterized fast‐cycling epidermal stem cells in young and aged mice (Figure 2F; Table S4; Raja et al. 2022). In 12‐month‐old *Fbln5* KO mice, the expression of epidermal basal markers was decreased, while genes associated with epidermal differentiation and hair follicle lineages were increased (Figure 2G; Table S4), consistent with features of accelerated epidermal stem cell differentiation and lineage impairment, as previously reported in aging mice (Raja et al. 2022). These results suggest that *Fbln5* KO mice exhibit global molecular changes resembling age‐dependent impairment of epidermal stem cell properties.

### 3.3 *Fbln5* Deficiency Compromises ECM Integrity at the Epidermal–Dermal Junction in Aging Skin

**FIGURE 3:**
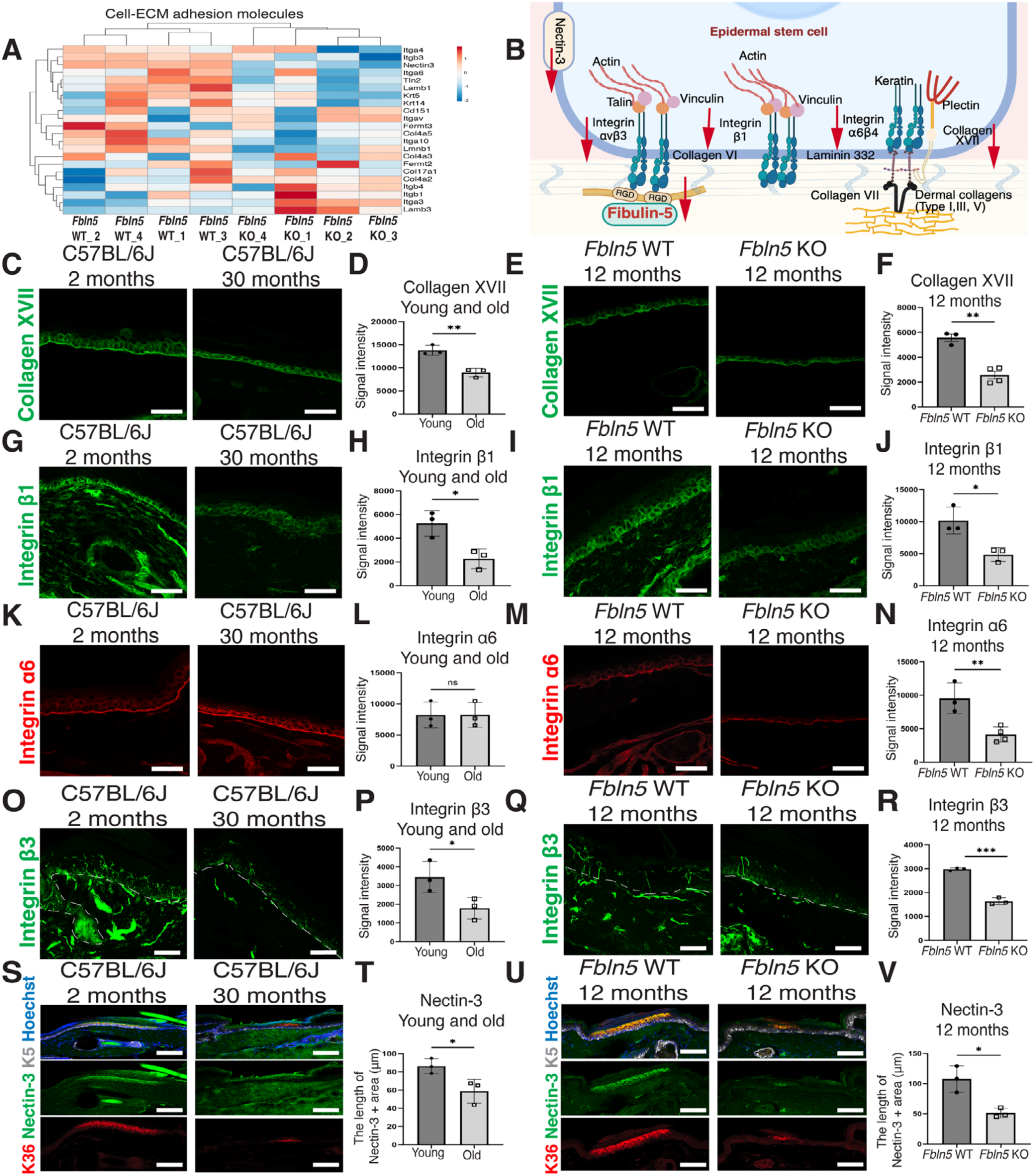
Changes in integrin and extracellular matrix expression due to the loss of fibulin‐5. (A) The heatmap shows changes in integrins and ECM proteins in 12‐month‐old *Fbln5* WT and KO epidermal stem cells. Genes with a ≥ 2‐fold change are used for analysis. (B) Schematic representation of the epidermal–dermal junction and its associated proteins. (C–V) Immunostaining and quantification of the indicated proteins: Collagen XVII (C–F; green), integrin β1 (G–J; green), integrin α6 (K–N; red) integrin β3 (O–R; green), nectin‐3 (S–V; green), K5 (S–V; gray), and K36 (S–V; red, scale lineage). The white dashed lines represent the epidermal–dermal boundary. Scale bars: 50 μm. All data are presented as the mean ± SD. Each dot represents one mouse. Statistical significance is assessed using a two‐tailed unpaired *t*‐test (D, F, H, J, N, P, R, T, V) or Mann–Whitney *U* test (L). *, *p* < 0.05; **, *p* < 0.01; ***, *p* < 0.001; ns, not significant. The schematic in panel B is created with BioRender.com.

Among the ECM proteins at the epidermal–dermal junction (Fujiwara 2024), the RNA‐seq data showed a trend of decreased expression for integrins, collagens, and laminins in the 12‐month‐old *Fbln5* KO mice (Figure 3A,B; Table S5). Immunostaining of these ECM proteins involved in epidermal stem cell regulation revealed that collagen XVII and integrin β1, which play essential roles in epidermal stem cell regulation (Brakebusch et al. 2000; Piwko‐Czuchra et al. 2009; Liu et al. 2019; Watanabe et al. 2017), were downregulated in both *Fbln5* KO and chronologically aged WT mice (Figure 3C–J). In contrast, integrin α6 was affected only in *Fbln5* KO mice (Figure 3K–N).

In the RNA‐seq data, we found significant downregulation of *Itgb3* and *Nectin3* (Figure S2B,E). Fibulin‐5 has been reported to interact with integrin αvβ3 via its RGD motif (Kobayashi et al. 2007; Nakamura et al. 2002; Yanagisawa et al. 2009). Nectin‐3 is an immunoglobulin‐like cell adhesion molecule that is located mainly at adherens junctions and plays a role in epidermal stratification (Mollo et al. 2015; Takahashi et al. 1999; Yoshida et al. 2014). It has been suggested that integrin αvβ3 and nectin‐3 interact through their extracellular regions and play roles in the crosstalk between cell–matrix and cell–cell junctions (Sakamoto et al. 2006; Takai et al. 2008). We examined the spatial localization of integrin β3 and nectin‐3 in young WT skin. Integrin β3 showed a fiber‐like pattern beneath the epidermis (Figure 3O), similar to the protein localization of fibulin‐5 (Figure 1B). Nectin‐3 was predominantly found in the upper layers at the scale region of the epidermis (Figure 3S). Integrin β3 and nectin‐3 expression were unaffected in 3‐month‐old *Fbln5* KO mice (Figure S2C,D,F,G). In aged mice and 12‐month‐old *Fbln5* KO mice, both integrin β3 and nectin‐3 were downregulated, consistent with a pattern of scale reduction observed with K36 (Figure 3O–V; Figure S2H,I). These results suggest that fibulin‐5 deficiency causes progressive alterations in integrin‐associated cell–matrix junction proteins and other adhesion molecules, which may contribute to age‐related epidermal stem cell dysfunction.

### 3.4 Reduced YAP Activity Is Associated With a Decline in Fast‐Cycling Stem Cells in *Fbln5* KO and Aged Skin

**FIGURE 4:**
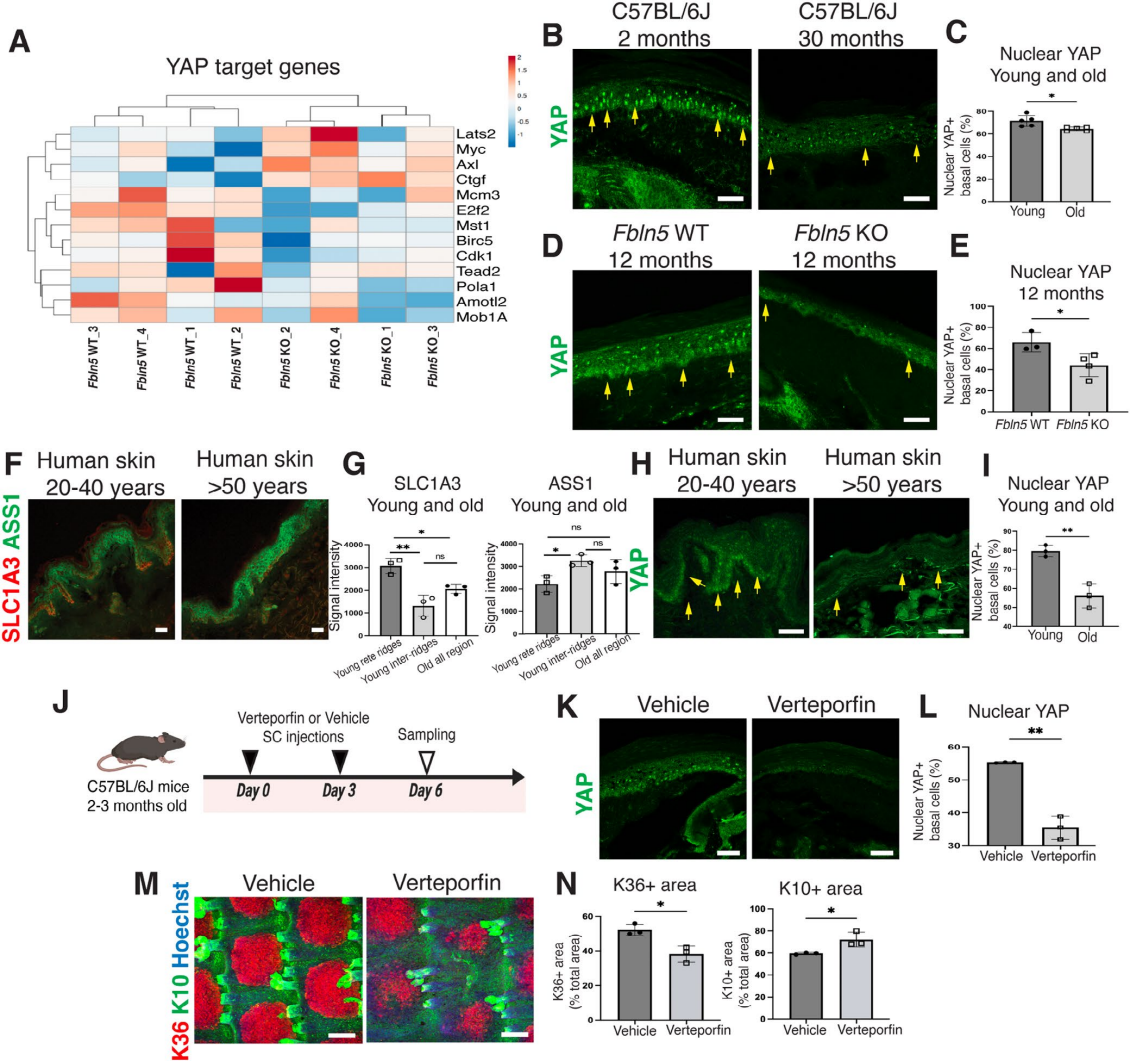
YAP downregulation is associated with a reduction in the fast‐cycling epidermal stem cell compartment. (A) The heatmap shows changes in YAP target gene expression in 12‐month‐old *Fbln5* WT and KO epidermal stem cells. Genes with a ≥ 2‐fold change are used for analysis. (B–E) Immunostaining of YAP (green) in sections of mouse tail skin and quantification (C, E). The yellow arrows indicate nuclear YAP signals. Nuclear YAP (%) was calculated as the proportion of cells with nuclear YAP localization among all Hoechst^+^ nuclei. Scale bars: 50 μm. (F, G) Immunostaining of ASS1 (green, slow‐cycling epidermal stem cells) and SLC1A3 (red, fast‐cycling epidermal stem cells) in sections of young (aged 20–40 years) and old (aged > 50 years) human skin and quantification (G). In young samples, basal cells are subdivided into rete ridge (0–40 μm) and inter‐ridge (> 40 μm) regions based on the distance along the basement membrane; in old samples, the entire basal layer is analyzed due to flattening of the rete ridge structures. Scale bars: 50 μm. (H, I) Immunostaining of YAP (green) in human skin and quantification (I). The yellow arrows indicate nuclear YAP signals. Scale bars: 50 μm. (J) Schematic representation of the verteporfin treatment assay. 2–3‐month‐old C57BL/6J mice receive subcutaneous injections of verteporfin or vehicle control on days 0 and 3 and are euthanized 3 days after the second injection (day 6) for tissue collection. (K, L) Immunostaining of YAP (green) and quantification (L). Scale bars: 50 μm. (M, N) Whole‐mount staining of K10 (green, interscale lineage), K36 (red, scale lineage), and Hoechst (blue), and quantification (N). Scale bars: 100 μm. All data are presented as the mean ± SD. Each dot represents one independent biological replicate. Statistical significance is assessed using a two‐tailed unpaired *t*‐test (C, E, I, N), Welch's *t*‐test (L), or one‐way ANOVA (G). *, *p* < 0.05; **, *p* < 0.01; ns, not significant. The schematic in panel J is created with BioRender.com.

Given the observed alterations in integrins and extracellular components in *Fbln5* KO and aged skin, we next examined whether intracellular signaling pathways regulating epidermal stem cells were affected, focusing on YAP signaling (Elbediwy et al. 2016; Lee et al. 2014; Schlegelmilch et al. 2011). In the RNA‐seq data, YAP‐associated cell cycle genes, such as *E2f2* and *Cdk1* (Pattschull et al. 2019), were downregulated in the *Fbln5* KO mice (Figure 4A, Figure S2A). Consistent with these transcriptional changes, immunostaining revealed reduced nuclear YAP localization in epidermal stem cells of *Fbln5* KO mice as well as chronologically aged WT mice (Figure 4B–E).

In humans, fibulin‐5 expression declines with age (Kadoya et al. 2005; Langton et al. 2019). Therefore, we investigated whether similar alterations in epidermal stem cell populations occur in human skin. The undulating rete ridge structure in human epidermis exhibits a spatially biased distribution of stem/progenitor markers (Ghuwalewala et al. 2022; Jensen et al. 1999; Legg et al. 2003; Wang et al. 2020; Webb et al. 2004; Dumrongphuttidecha et al. 2025). In young human skin (aged 20–40 years), we found that SLC1A3, a marker of the fast‐cycling population identified in mice and humans (Ghuwalewala et al. 2022; Sada et al. 2016; Dumrongphuttidecha et al. 2025), showed significantly higher fluorescence intensity in basal cells within the 0–40 μm rete ridge compartment, whereas ASS1 fluorescence intensity was more enriched in basal cells located in the > 40 μm inter‐ridge compartment (Figure 4F,G). In aged human skin (> 50 years), the rete ridge structure was markedly flattened, as previously reported (Giangreco et al. 2010). Under these conditions, SLC1A3 intensity was significantly reduced, whereas ASS1 expression was broadly expanded across the basal layer (Figure 4F,G). Consistent with these changes, nuclear YAP in the epidermal basal layer was also significantly decreased in aged human skin (Figure 4H,I). These results indicate that aging in human epidermis is associated with reduced YAP activity and a global shift from an SLC1A3‐enriched fast‐cycling state toward an ASS1‐enriched phenotype. These changes resemble those observed in chronologically aged WT mice and *Fbln5* KO mice, which exhibited a loss of scale (fast‐cycling) regions (Figure 1P,Q,T,U) and inactivation of YAP signaling (Figure 4B–E).

To functionally test whether YAP activity contributes to the decline of fast‐cycling stem cell populations in vivo, we pharmacologically inhibited YAP signaling in WT mice by subcutaneous injection of verteporfin into the tail skin (Figure 4J). Verteporfin treatment significantly reduced nuclear YAP localization in the epidermal basal cells (Figure 4K,L), confirming effective YAP inhibition. Notably, verteporfin treatment significantly reduced the K36^+^ scale (fast‐cycling) area and concomitantly increased the K10^+^ interscale (slow‐cycling) area (Figure 4M,N), phenocopying the imbalance observed in *Fbln5* KO or aged WT mice (Figure 1P,Q,T,U). These findings support a role for YAP activity in maintaining the fast‐cycling epidermal stem cell compartment in vivo.

### 3.5 Extracellular Fibulin‐5 Enhances YAP‐Dependent Fast‐Cycling Stem Cell Signatures in Human Keratinocytes

**FIGURE 5:**
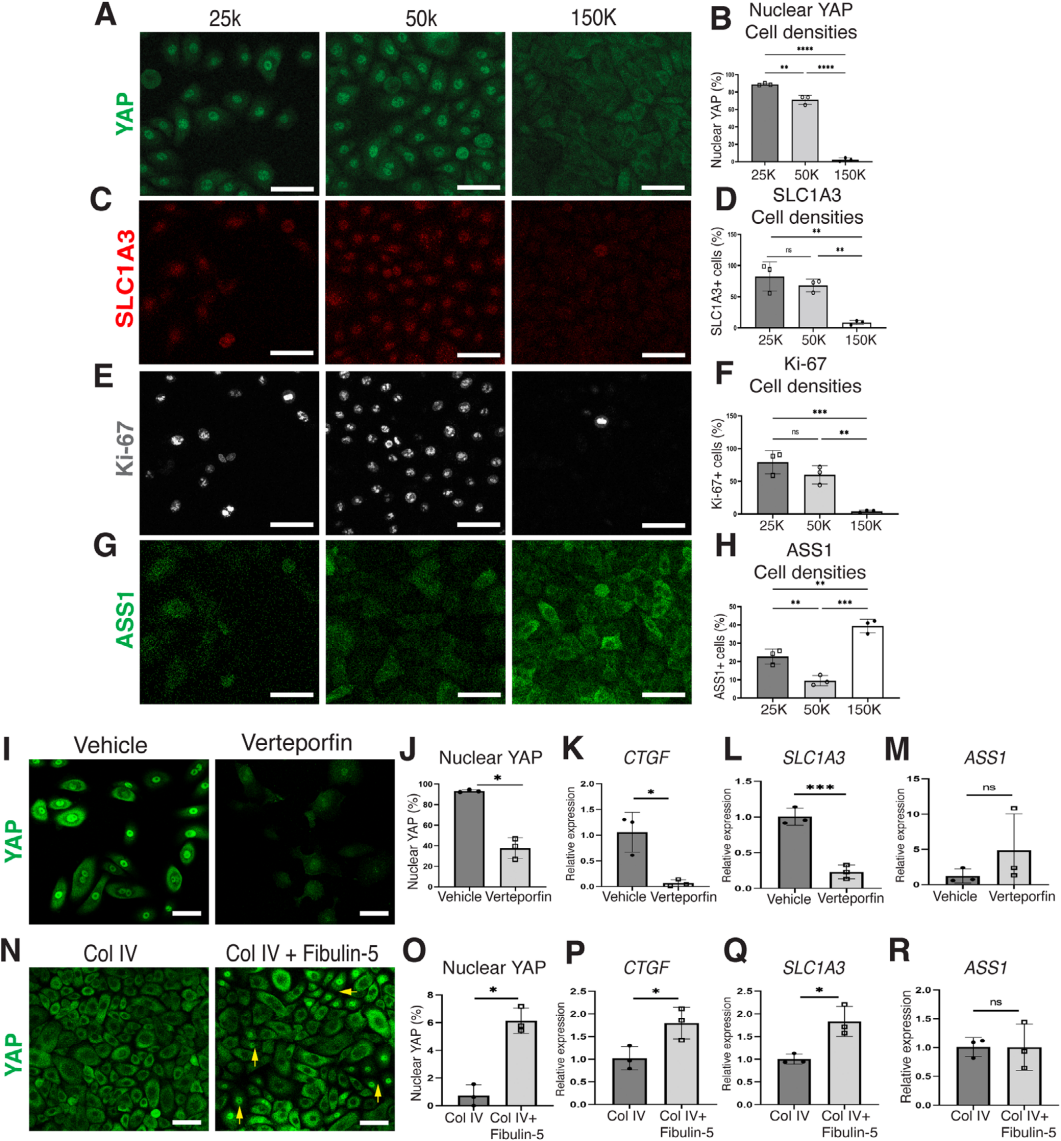
Extracellular fibulin‐5 enhances YAP activity and fast‐cycling stem cell‐associated gene expression in human keratinocytes. (A–H) Immunostaining of YAP (A, green), SLC1A3 (C, red), Ki‐67 (E, gray), and ASS1 (G, green) in human keratinocytes and quantification (B, D, F, H). Cells are seeded at 150,000, 50,000, and 25,000 cells per well in 12‐well plates and cultured for 48 h before analysis. Scale bars: 50 μm. (I, J) Immunostaining of YAP in primary human keratinocytes and quantification (J). Cells are seeded at 50,000 cells per well in 12‐well plates and cultured for 24 h and then treated with verteporfin or vehicle control for 8 h. Nuclear YAP (%) was calculated as the proportion of cells with nuclear YAP localization among all Hoechst^+^ nuclei. Scale bars: 50 μm. (K–M) RT‐qPCR analysis of *CTGF*, *SLC1A3*, and *ASS1* following 8 h of verteporfin treatment. (N, O) Immunostaining of YAP in primary human keratinocytes and quantification (O). Cells are seeded at 300,000 cells per well on collagen IV–coated plates with or without recombinant human fibulin‐5 and cultured to ~80% confluence. The medium is then replaced, and cells are analyzed 8 h later. Scale bars: 50 μm. (P–R) RT‐qPCR analysis of *CTGF*, *SLC1A3*, and *ASS1* following culture on plates coated with collagen IV ± fibulin‐5. All data are presented as the mean ± SD. Each dot represents one independent biological replicate. Statistical significance is assessed using a two‐tailed unpaired *t*‐test (K, L, P, Q, R), Welch's *t*‐test (J, M, O), or one‐way ANOVA (B, D, F, H). *, *p* < 0.05; **, *p* < 0.01; ***, *p* < 0.001; ****, *p* < 0.0001; ns, not significant.

To examine whether YAP activity directly influences the balance of the epidermal stem cell population, we employed an in vitro cell‐density model to modulate YAP activity (Panciera et al. 2017). Culturing human primary keratinocytes (epidermal stem/progenitor cells) at a higher density inactivated YAP activity and increased early differentiation markers, while epidermal basal markers remained unchanged (Figure 5A,B; Figure S3A,B). Under these conditions, SLC1A3 (a fast‐cycling epidermal stem cell or rete ridge marker) and Ki‐67 (a proliferation marker) were downregulated, while ASS1 (an inter‐ridge marker) was upregulated (Figure 5C–H; Figure S3C). To further test this relationship, we pharmacologically inhibited YAP using verteporfin in primary human keratinocytes. Verteporfin treatment for 8 h reduced nuclear YAP localization and *CTGF* expression (Figure 5I–K), confirming the YAP inhibition. Consistent with this, *SLC1A3* expression decreased, whereas *ASS1* expression showed a modest increasing trend (Figure 5L,M). These results suggest that YAP suppression is associated with a downregulation of the fast‐cycling stem cell‐associated phenotype.

Finally, we investigated whether extracellular fibulin‐5 could modulate YAP signaling. Primary human keratinocytes were cultured on plates coated with recombinant fibulin‐5 under high‐density conditions, in which YAP activity is suppressed. The fibulin‐5 coating partially restored nuclear YAP localization and significantly increased *SLC1A3* and *CTGF* expression (Figure 5N–R), suggesting that fibulin‐5 enhances YAP signaling and supports a fast‐cycling status. However, fibulin‐5 coating alone was insufficient to induce *ITGAV*, *ITGB1*, *NECTIN3*, *ITGA6*, or *COL17A1* mRNA expression (Figure S3D), suggesting that fibulin‐5 enhances YAP signaling without directly altering integrin or other ECM gene expression at the transcriptional level. Together, these data support a model in which extracellular fibulin‐5 contributes to the regulation of YAP activity and fast‐cycling stem cell‐associated gene expression in human keratinocytes, consistent with the in vivo observations.

## 4 Discussion

**FIGURE 6:**
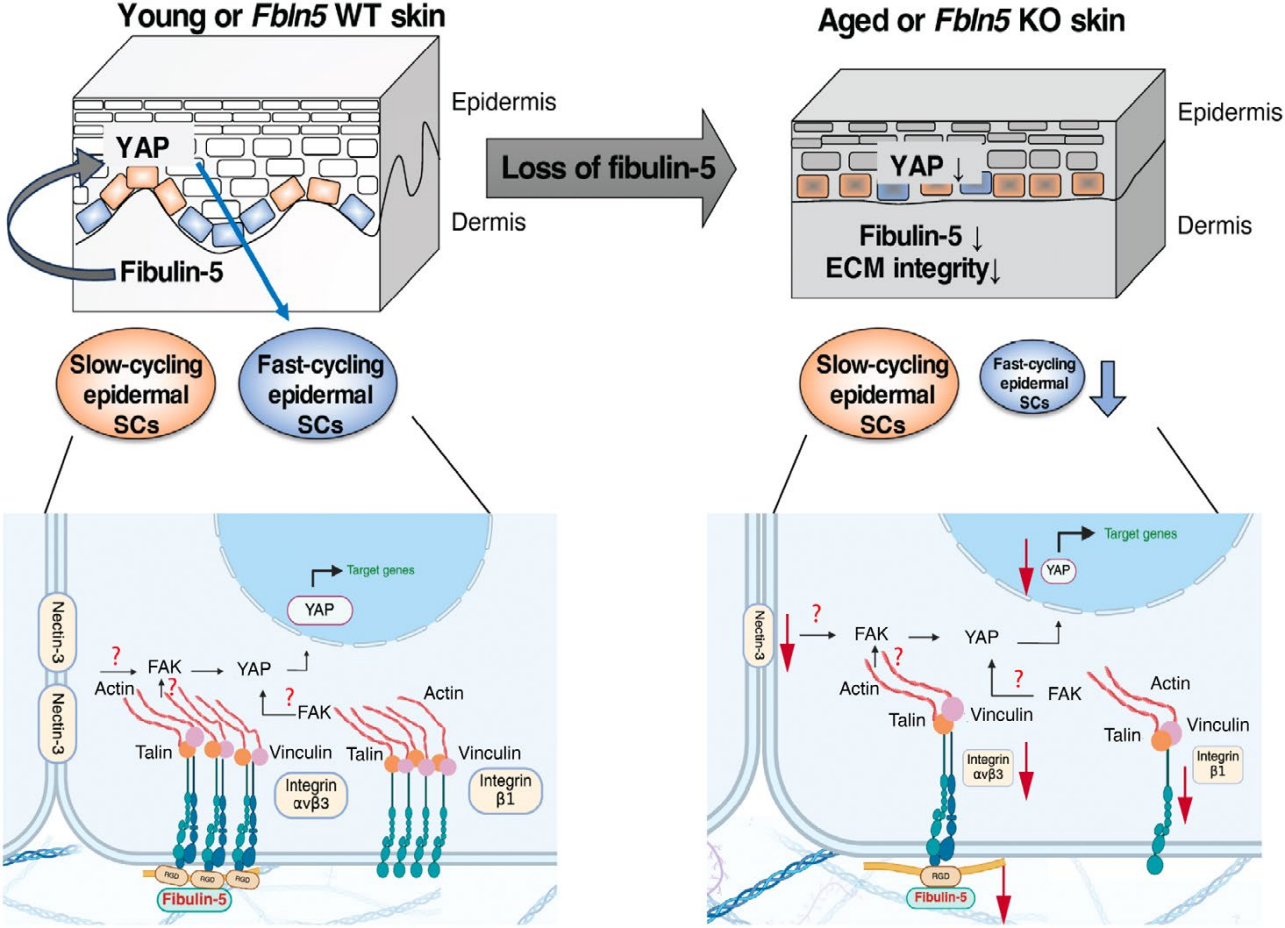
Proposed model of cellular and molecular alterations associated with fibulin‐5 deficiency during skin aging. In young skin, slow‐cycling and fast‐cycling epidermal stem cells (SCs) are spatially compartmentalized and give rise to their respective lineages. During aging, decreased fibulin‐5 expression is associated with altered integrin and extracellular matrix (ECM) protein expression, potentially affecting intracellular signaling through fibulin‐5–integrin interactions. Reduced YAP activity is associated with a decrease in the fast‐cycling epidermal stem cell compartment in aged skin and human keratinocytes. The schematic is created with BioRender.com.

The environment surrounding epidermal stem cells changes during skin aging, yet the mechanisms linking extracellular changes to intracellular responses remain unclear. In this study, we found that fibulin‐5, a matricellular protein whose expression decreases with skin aging, plays a role in maintaining epidermal stem cell heterogeneity (Figure 6). Although fibulin‐5 has been extensively studied for its role in elastic fiber formation, our findings suggest an additional role in shaping the epidermal stem cell niche during aging. Because the loss of fibulin‐5 is also seen in chronological aging (Kadoya et al. 2005), photoaging (Langton et al. 2019), and cutis laxa (Gharesouran et al. 2021), fibulin‐5–mediated regulation of the epidermal stem cell environment may have potential relevance to human skin aging.

The loss of fibulin‐5 is associated with progressive alterations in ECM composition and structure, accompanied by impairments in cellular and molecular properties of epidermal stem cells over time. While compensatory mechanisms may maintain ECM integrity at a young age, these mechanisms remain unclear. As the mice age, there may be combinatorial changes in the ECM, accumulation of DNA damage and mutations, and a chronic inflammatory environment in their skin (Jurk et al. 2014; Raja et al. 2022) that exacerbate the changes caused by fibulin‐5 deficiency.

While the mechanisms through which fibulin‐5 controls epidermal stem cell heterogeneity remain unclear, prior studies suggest functional links between fibulin‐5, integrin, and YAP signaling pathways. Fibulin‐5 contains an RGD motif and has been reported to interact with integrins, including αvβ3 and αvβ5, and potentially with β1 integrins (Lomas et al. 2007; Nakamura et al. 2002; Yanagisawa et al. 2009; Schluterman et al. 2010). These integrins act as mediators of matrix mechanotransduction, with α5β1 contributing to adhesion strength and αvβ3 participating in the early stages of force sensing (Roca‐Cusachs et al. 2009). Integrin‐dependent FAK signaling promotes YAP activation in skin (Elbediwy et al. 2016) and other tissues (Yamashiro et al. 2020).

In the present study, we observed changes in the ECM composition, integrin expression, and YAP activity in *Fbln5* KO and chronologically aged WT skin. Among integrins, integrin β1, a key regulator of epidermal stem cells, declines in aged human epidermis (Giangreco et al. 2010), and *Itgb1* KO mice exhibit loss of the scale region (López‐Rovira et al. 2005), similar to that seen in *Fbln5* KO mice. In parallel, cell–cell adhesion proteins also play essential roles in regulating epidermal patterning and YAP activity (Mai et al. 2024). Integrin β3, associated with nectin‐3, may influence cell–cell junctional stability and the nuclear translocation of YAP in vitro (Nardone et al. 2017; Stanton et al. 2019). Integrin α6 was selectively reduced in *Fbln5* KO skin but not in chronological aging, which suggests that fibulin‐5 deficiency may uniquely affect basement membrane integrity. These differential patterns suggest that integrins β1, β3, and α6 play distinct roles in the adhesive and mechanosensitive regulation of YAP signaling; however, our data can only show a correlation rather than direct mechanistic causality in the fibulin‐5–integrin–YAP axis. Future biochemical and genetic approaches will be required to determine integrin specificity and a direct mechanistic relationship between fibulin‐5 and YAP signaling for the maintenance of the fast‐cycling stem cell compartment during aging.

An additional consideration in interpreting the in vivo phenotype relates to the structural role of fibulin‐5 in elastic fiber assembly. *Fbln5* deficiency disrupts elastic fiber formation and increases skin laxity (Yanagisawa et al. 2002), potentially altering the mechanical properties, such as stiffness and tensile stress. Thus, decreased YAP activity in *Fbln5* KO skin may also reflect altered tissue mechanics independently of the direct fibulin‐5–integrin axis.

Collectively, our findings identify fibulin‐5 as a niche‐associated ECM component whose age‐dependent decline is associated with changes in extracellular organization, YAP activity, and epidermal stem cell balance. Our study suggests that fibulin‐5 reduction may alter adhesive and potentially mechanical aspects of the skin microenvironment, thereby influencing intracellular signaling states in epidermal stem cells.

## Author Contributions

Aiko Sada conceptualized the project, designed the experiments, and contributed expertise in stem cell and skin biology. Wenxin Fan, Mizuho Ishikawa, Erna Raja, Ahmed M. Hegazy, Hinata Date, Yen Xuan Ngo, and Yoshifumi Sato performed the experiments and analyzed the data. Hiromi Yanagisawa provided the *Fbln5* KO mice and fibulin‐5 antibodies and contributed expertise on the ECM. Yoshifumi Sato and Kazuya Yamagata provided the aged C57BL/6J mice. Aiko Sada managed and supervised the project. The manuscript was drafted by Wenxin Fan and edited by Aiko Sada, Erna Raja, Mizuho Ishikawa, and Hiromi Yanagisawa. Funding was acquired by Aiko Sada.

## Funding

This work was supported by Japan Agency for Medical Research and Development, JP23bm0704067, JP21gm6110016, JP22jm0610063. Japan Society for the Promotion of Science, JP20H03266, JP24K02035. Uehara Memorial Foundation. Japan Science and Technology Agency, JPMJSP2127. Takeda Science Foundation.

## Disclosure

Declaration of Generative AI and AI‐Assisted Technologies in the Writing Process: While preparing this manuscript, the authors used ChatGPT to assist with language clarity and readability. Following these tools, the authors thoroughly reviewed and edited the content to ensure its accuracy and take full responsibility for the final version of the published article.

## Conflicts of Interest

The authors declare no conflicts of interest.

## Supporting information


**Figure S1:** Phenotype analysis of *Fbln5* KO mice at different time points. (A, B) Hematoxylin and eosin staining of sagittal sections of the skin of 6‐month‐old *Fbln5* WT and KO mice and quantification (B). Scale bars: 150 μm. Epidermal thickness was measured in interscale and scale regions. (C–F) Whole‐mount staining of BrdU (C, green), Hoechst (C, blue), and Ki‐67 (E, gray) in 6‐ and 12‐month‐old *Fbln5* WT and KO mice and quantification (D, F). Scale bars: 200 μm. (G, H) Whole‐mount staining of K10 (green, interscale lineage), K36 (red, scale lineage), and Hoechst (blue) from 2‐week‐old *Fbln5* WT and KO mice and quantification (H). Scale bars: 200 μm. (I, J) Whole‐mount staining of K10 (green, interscale lineage) and K36 (red, scale lineage) from 6‐month‐old *Fbln5* WT and KO mice and quantification (J). Scale bars: 200 μm. All data are presented as the mean ± SD. Each dot represents one mouse. Statistical significance is assessed using a two‐tailed unpaired *t*‐test (B, D, F, H, J). *, *p* < 0.05; ns, not significant.
**Figure S2:** Expression of integrin β3 and nectin‐3 in *Fbln5* KO mice. (A) RT qPCR analysis of *Nectin3*, *E2f2*, and *Cdk1* expression in FACS‐sorted epidermal stem cells from 12‐month‐old *Fbln5* WT and KO mice. (B) RNA‐seq analysis of *Itgb3* gene expression in 12 month‐old *Fbln5* WT and KO epidermal stem cells. (C, D) Immunostaining of integrin β3 (green) and quantification (D). The white dashed line represents the epidermal–dermal boundary. Scale bars: 20 μm. (E) RNA‐seq analysis of *Nectin3* gene expression in 12‐month‐old *Fbln5* WT and KO epidermal stem cells. (F–I) Immunostaining of nectin‐3 (green), K36 (red, scale lineage), K5 (gray, basal layer), and Hoechst (blue), and quantification (G–I). The length of the nectin‐3^+^ area (G) and the overlapping signal of nectin‐3 in K36^+^ regions (H, I) are quantified. Scale bars: 50 μm. All data are presented as the mean ± SD. Each dot represents one mouse. Statistical significance is assessed using a two‐tailed unpaired *t*‐test (A, B, D, E, G, H, I). *, *p* < 0.05; **, *p* < 0.01; ****, *p* < 0.0001; ns, not significant.
**Figure S3:** RT‐qPCR analysis of human keratinocytes. (A–C) RT‐qPCR analysis of epidermal marker genes in primary human keratinocytes cultured at different densities. Cells are seeded at 150,000, 50,000, and 25,000 cells per well in 12‐well plates and cultured for 48 h before analysis. (D) RT‐qPCR analysis of *ITGAV*, *ITGB1*, *NECTIN3*, *ITGA6*, and *COL17A1* following culture on plates coated with collagen IV ± fibulin‐5. All data are presented as the mean ± SD. Each dot represents one independent biological replicate. Statistical significance is assessed using a one‐way ANOVA (A–C), an unpaired *t*‐test (D, *ITGB1*, *ITGA6*), Welch's *t*‐test (D, *COL17A1*), or a Mann–Whitney *U* test (D, *ITGAV*, *NECTIN3*). *, *p* < 0.05; ns, not significant.
**Table S1:** Donor information for human skin samples.
**Table S2:** Primers used for RT‐qPCR.

**Table S3:** List of differentially expressed genes identified by RNA‐seq analysis of epidermal basal cells from *Fbln5* WT and KO mice (related to Figure 2C).

**Table S4:** List of genes associated with epidermal lineage and differentiation programs used to analyze the RNA‐seq datasets of *Fbln5* WT and KO epidermal basal cells and fast‐cycling epidermal stem cells (non‐label‐retaining cells) from young and old WT mice (related to Figure 2F,G).

**Table S5:** List of extracellular matrix (ECM) and YAP‐related genes showing altered expression in epidermal basal cells from *Fbln5* WT and KO mice (related to Figures 3A and 4A).

## Data Availability

The authors declare that the data supporting the findings of this study are available within the paper and its Supporting Information files. The RNA‐seq data are deposited in the Gene Expression Omnibus (accession number: GSE295189).
