# Supplementary material for: Integrin‐Binding Matricellular Protein Fibulin‐5 Maintains Epidermal Stem Cell Heterogeneity During Skin Aging
Source: Aging Cell. 2026 Apr 10;25(4):e70483. doi: 10.1111/acel.70483 (PMC13067917; doi:10.1111/acel.70483)
Supplement: Supplementary file 1 — Figure S1: Phenotype analysis of Fbln5 KO mice at different time points. (A, B) Hematoxylin and eosin staining of sagittal sections of the skin of 6‐month‐old Fbln5 WT and KO mice and quantification (B). Scale bars: 150 μm. Epidermal thickness was measured in interscale and scale regions. (C–F) Whole‐mount staining of BrdU (C, green), Hoechst (C, blue), and Ki‐67 (E, gray) in 6‐ and 12‐month‐old Fbln5 WT and KO mice and quantification (D, F). Scale bars: 200 μm. (G, H) Whole‐mount staining of K10 (green, interscale lineage), K36 (red, scale lineage), and Hoechst (blue) from 2‐week‐old Fbln5 WT and KO mice and quantification (H). Scale bars: 200 μm. (I, J) Whole‐mount staining of K10 (green, interscale lineage) and K36 (red, scale lineage) from 6‐month‐old Fbln5 WT and KO mice and quantification (J). Scale bars: 200 μm. All data are presented as the mean ± SD. Each dot represents one mouse. Statistical significance is assessed using a two‐tailed unpaired t‐test (B, D, F, H, J). *, p < 0.05; ns, not significant. Figure S2: Expression of integrin β3 and nectin‐3 in Fbln5 KO mice. (A) RT qPCR analysis of Nectin3, E2f2, and Cdk1 expression in FACS‐sorted epidermal stem cells from 12‐month‐old Fbln5 WT and KO mice. (B) RNA‐seq analysis of Itgb3 gene expression in 12 month‐old Fbln5 WT and KO epidermal stem cells. (C, D) Immunostaining of integrin β3 (green) and quantification (D). The white dashed line represents the epidermal–dermal boundary. Scale bars: 20 μm. (E) RNA‐seq analysis of Nectin3 gene expression in 12‐month‐old Fbln5 WT and KO epidermal stem cells. (F–I) Immunostaining of nectin‐3 (green), K36 (red, scale lineage), K5 (gray, basal layer), and Hoechst (blue), and quantification (G–I). The length of the nectin‐3+ area (G) and the overlapping signal of nectin‐3 in K36+ regions (H, I) are quantified. Scale bars: 50 μm. All data are presented as the mean ± SD. Each dot represents one mouse. Statistical significance is assessed using a two‐tailed unpaired t‐test ( [file ACEL-25-e70483-s002.pdf]

Supplemental Information

Supplementary Figure 1

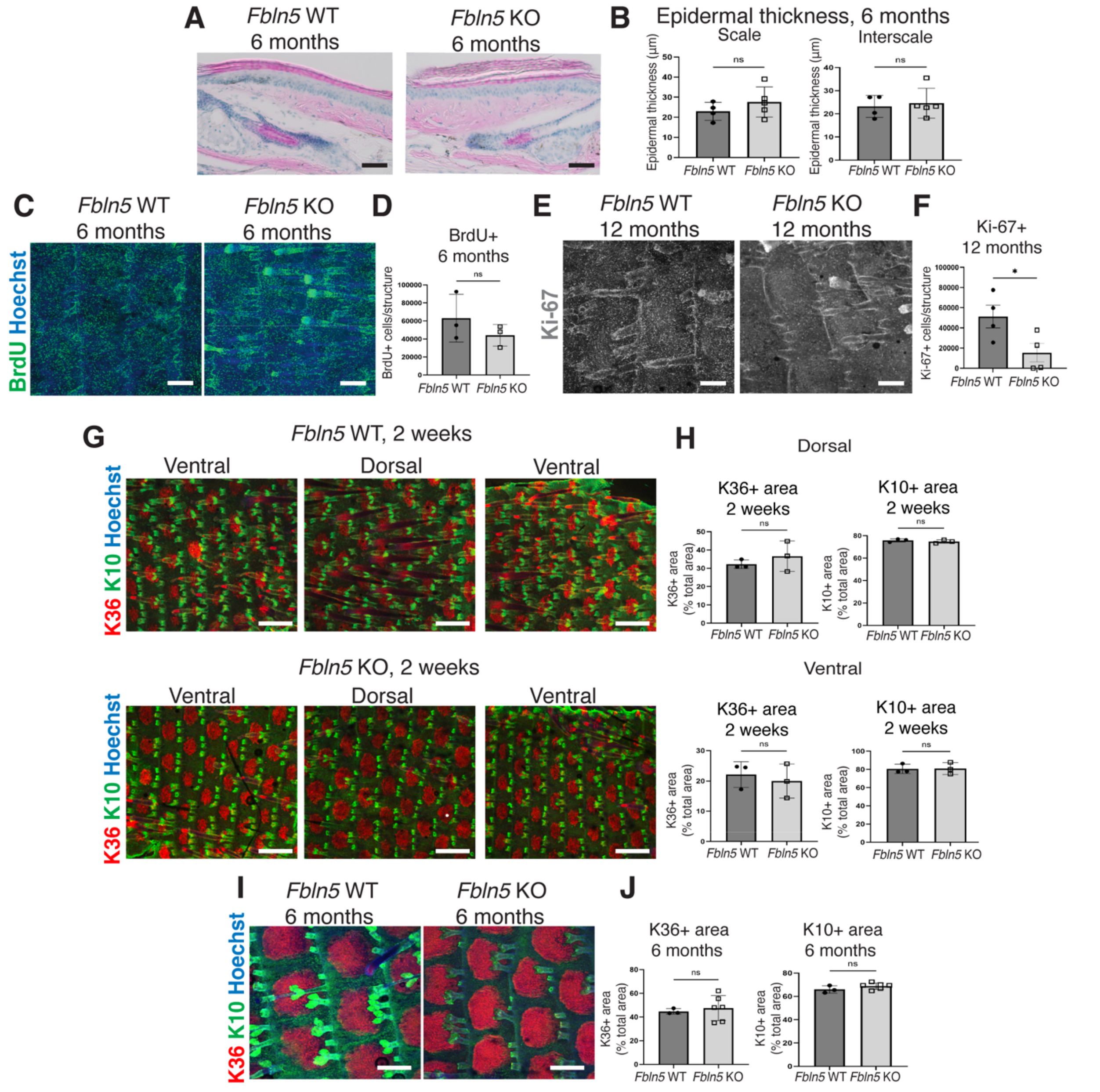

Supplementary Figure S1. Phenotype analysis of *Fbln5* KO mice at different time points. (**A, B**) Hematoxylin and eosin staining of sagittal sections of the skin of 6-month-old *Fbln5* WT and KO mice and quantification (B). Scale bars: 150 μm. Epidermal thickness was measured in interscale and scale regions. (**C–F**) Whole-mount staining of BrdU (C, green), Hoechst (C, blue), and Ki-67 (E, gray) in 6- and 12-month-old *Fbln5* WT and KO mice and quantification (D, F). Scale bars: 200 μm. (**G, H**) Whole-mount staining of K10 (green, interscale lineage), K36 (red,

scale lineage), and Hoechst (blue) from 2-week-old *Fbln5* WT and KO mice and quantification (H). Scale bars: 200 μm. (**I, J**) Whole-mount staining of K10 (green, interscale lineage) and K36 (red, scale lineage) from 6-month-old *Fbln5* WT and KO mice and quantification (J). Scale bars: 200 μm. All data are presented as the mean ± SD. Each dot represents one mouse. Statistical significance is assessed using a two-tailed unpaired t-test (B, D, F, H, J). *, $p < 0.05$; ns, not significant.

Supplementary Figure 2

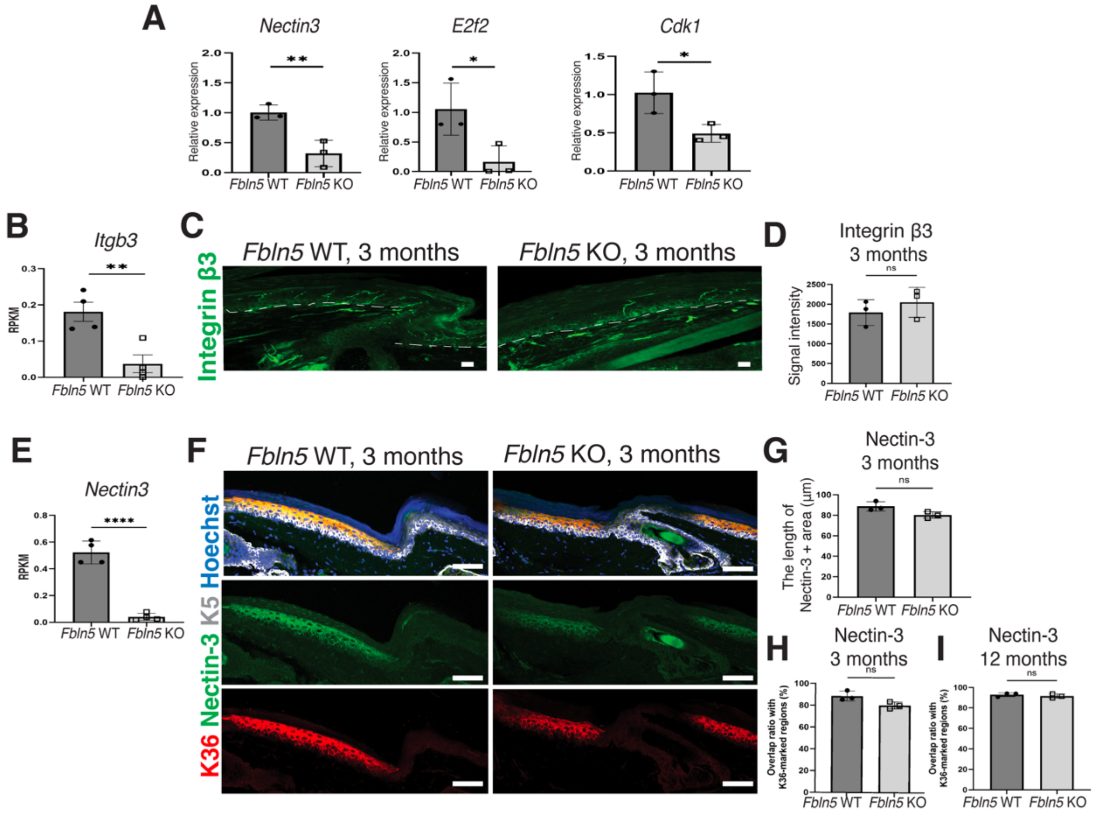

Supplementary Figure S2. Expression of integrin β3 and nectin-3 in *Fbln5* KO mice. (**A**) RT-qPCR analysis of *Nectin3*, *E2f2*, and *Cdk1* expression in FACS-sorted epidermal stem cells from 12-month-old *Fbln5* WT and KO mice. (**B**) RNA-seq analysis of *Itgb3* gene expression in 12-month-old *Fbln5* WT and KO epidermal stem cells. (**C**, **D**) Immunostaining of integrin β3 (green) and quantification (D). The white dashed line represents the epidermal–dermal boundary. Scale bars: 20 µm. (**E**) RNA-seq analysis of *Nectin3* gene expression in 12-month-old *Fbln5* WT and KO epidermal stem cells. (**F**–**I**) Immunostaining of nectin-3 (green), K36 (red, scale lineage), K5 (gray, basal layer), and Hoechst (blue), and quantification (G–I). The length of the nectin-3$^+$ area (G) and the overlapping signal of nectin-3 in K36$^+$ regions (H, I) are quantified. Scale bars: 50 µm. All data are presented as the mean ± SD. Each dot represents one mouse. Statistical significance is assessed using a two-tailed unpaired t-test (A, B, D, E, G, H, I). *, $p < 0.05$; **, $p < 0.01$; ****, $p < 0.0001$; ns, not significant.

Supplementary Figure 3

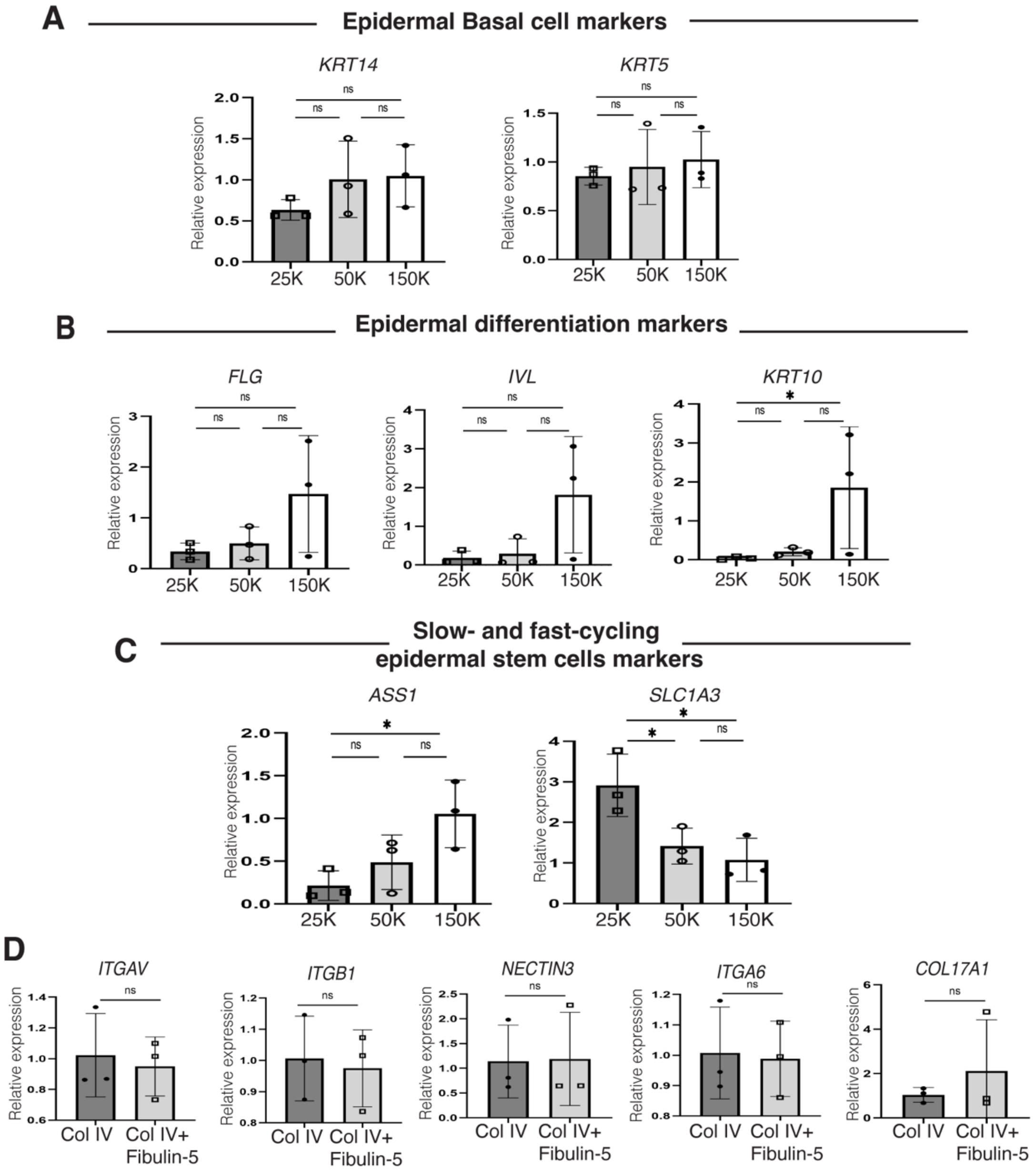

Supplementary Figure S3. RT-qPCR analysis of human keratinocytes. (**A**–**C**) RT-qPCR analysis of epidermal marker genes in primary human keratinocytes cultured at different densities. Cells are seeded at 150,000, 50,000, and 25,000 cells per well in 12-well plates and cultured for 48 hours before analysis. (**D**) RT-qPCR analysis of *ITGAV*, *ITGB1*, *NECTIN3*, *ITGA6*, and *COL17A1* following culture on plates coated with collagen IV ± fibulin-5. All data are presented as the mean ± SD. Each dot represents one independent biological replicate. Statistical significance is assessed using a one-way ANOVA (A–C), an unpaired t-test (D, *ITGB1, ITGA6*), Welch's t-test (D, *COL17A1*), or a Mann-Whitney U test (D, *ITGAV, NECTIN3*). *, $p < 0.05$; ns, not significant.

Supplementary Table 1: Donor information for human skin samples

Category	ID	Age	Sex	Skin part	Ethnicity
Young	SK0163	25 years	F	Abdomen	N/A
Young	SK0548	29 years	F	Abdomen	Caucasian
Young	SK0550	38 years	F	Abdomen	Caucasian
Old	PK003	74 years	M	Abdomen	N/A
Old	SK0033	63 years	F	Abdomen	N/A
Old	SK0220	52 years	F	Abdomen	N/A

Supplementary Table 2: Primers used for RT-qPCR

Species	Gene Symbol	Primer	Sequence
Human	KRT14	forward	AGATGATTGGCAGCGTGGAG
		reverse	AACTGGGAGGAGGAGAGGTG
	KRT5	forward	TGCTGAGAATGAGTTTGTGATGC
		reverse	AGGTTGCGGTTGTTGTCCAT
	FLG	forward	CAGTGAGGCATACCCAGAGG
		reverse	ACTGGCTGTATCGCGGTGAG
	IVL	forward	GAACAGCAGGAAAAGCACCT
		reverse	CTGGTTGAATGTCTTGGACCT
	KRT10	forward	TCGGGCTCTGGAAGAATCAA
		reverse	CTGAAGCAGGATGTTGGCATT
	ASS1	forward	TCTACAACCGGTTCAAGGGC
		reverse	TCCAGGATTCCAGCCTCGTA
	SLC1A3	forward	AGCAGGGAGTCCGTAAACGC
		reverse	TGGTCGGAGGGTAAATCCAAGG
	CTGF	forward	AACCAGCCCAAACAGAACCA
		reverse	TCCTCTTCCTCTCCCTCTGC
	ITGAV	forward	CGCTTCTTCTCTCGGGACTC
		reverse	CTGGGTGGTGTTTGCTTTGG
	ITGB1	forward	CCTACTTCTGCACGATGTGATG
		reverse	CCTTTGCTACGGTTGGTTACATT
	NECTIN3	forward	GCAGTTCACCATCCCCAATATG
		reverse	TCCAAGCGGGAATGTAACAGC
	ITGA6	forward	CACATCTCCTCCCTGAGCAC
		reverse	TATCTTGCCACCCATCCTTG
	COL17A1	forward	GAGATGGGTCCCTCCTGTCT
		reverse	AGGAAGCCCAATGCTGATCC

	ACTB	forward	GATCAGCAAGCAGGAGTACGA
		reverse	AAAACGCAGCTCAGTAACAGTC
	GAPDH	forward	ACTGCCACCCAGAAGACTGT
		reverse	GATGCAGGGATGATGTTCTG
Mouse	Nectin3	forward	GTAGCAGCCGTTTGTGTAGCA
		reverse	AGGTGAGGAATGCCACTGAAC
	E2f2	forward	CTGAATTCCGGACCCCCAAG
		reverse	CGACGTGTCATAGCGTGTCT
	Cdk1	forward	CTCGGCTCGTTACTCCACTC
		reverse	ACTCGACTTCTGGCCACACT
	Actb	forward	AGAGATGGCCACGGCTGCTT
		reverse	ATTTGCGGTGGACGATGGAG

Supplementary Table 3: List of differentially expressed genes identified by RNA-seq analysis of epidermal basal cells from *Fbln5* WT and KO mice (related to Fig. 2C).

Supplementary Table 4: List of genes associated with epidermal lineage and differentiation programs used to analyze the RNA-seq datasets of *Fbln5* WT and KO epidermal basal cells and fast-cycling epidermal stem cells (non-label-retaining cells) from young and old WT mice (related to Fig. 2F, G).

Supplementary Table 5: List of extracellular matrix (ECM) and YAP-related genes showing altered expression in epidermal basal cells from *Fbln5* WT and KO mice (related to Fig. 3A, 4A).
